# Fetal brain genomic reprogramming following asphyctic preconditioning

**DOI:** 10.1186/1471-2202-14-61

**Published:** 2013-06-22

**Authors:** Kimberly EM Cox-Limpens, Johan SH Vles, Jana Schlechter, Luc JI Zimmermann, Eveline Strackx, Antonio WD Gavilanes

**Affiliations:** 1 School for Mental Health and Neuroscience (MHeNS), Maastricht University, Universiteitssingel 50, 6200, MD Maastricht, The Netherlands; 2 Department of Pediatrics, Maastricht University Medical Center (MUMC), Postbus 5800, Maastricht, AZ, 6202, The Netherlands; 3 Department of Pediatric Neurology, Maastricht University Medical Center (MUMC), P.Debyelaan 25, 6229, HX Maastricht, The Netherlands

**Keywords:** Asphyxia, Epigenetic, Fetal brain, Hypoxia-ischemia, Micro-array, Preconditioning

## Abstract

**Background:**

Fetal asphyctic (FA) preconditioning is effective in attenuating brain damage incurred by a subsequent perinatal asphyctic insult. Unraveling mechanisms of this endogenous neuroprotection, activated by FA preconditioning, is an important step towards new clinical strategies for asphyctic neonates. Genomic reprogramming is thought to be, at least in part, responsible for the protective effect of preconditioning. Therefore we investigated whole genome differential gene expression in the preconditioned rat brain. FA preconditioning was induced on embryonic day 17 by reversibly clamping uterine circulation. Male control and FA offspring were sacrificed 96 h after FA preconditioning. Whole genome transcription was investigated with Affymetrix Gene1.0ST chip.

**Results:**

Data were analyzed with the Bioconductor Limma package, which showed 53 down-regulated and 35 up-regulated transcripts in the FA-group. We validated these findings with RT-qPCR for *adh1*, *edn1*, *leptin*, *rdh2*, and *smad6*. Moreover, we investigated differences in gene expression across different brain regions. In addition, we performed Gene Set Enrichment Analysis (GSEA) which revealed 19 significantly down-regulated gene sets, mainly involved in neurotransmission and ion transport. 10 Gene sets were significantly up-regulated, these are mainly involved in nucleosomal structure and transcription, including genes such as *mecp2*.

**Conclusions:**

Here we identify for the first time differential gene expression after asphyctic preconditioning in fetal brain tissue, with the majority of differentially expressed transcripts being down-regulated. The observed down-regulation of cellular processes such as neurotransmission and ion transport could represent a restriction in energy turnover which could prevent energy failure and subsequent neuronal damage in an asphyctic event. Up-regulated transcripts seem to exert their function mainly within the cell nucleus, and subsequent Gene Set Enrichment Analysis suggests that epigenetic mechanisms play an important role in preconditioning induced neuroprotection.

## Background

Hypoxic-ischemia or asphyxia, whether it occurs pre-, peri- or postnatally, is still a major cause of neonatal mortality and morbidity. It is frequently associated with permanent neurological deficits, such as motor disabilities, learning and cognitive problems. Nowadays, post-asphyctic hypothermia is the only available evidence-based therapeutic strategy for treating term asphyxiated infants. However, only a subset of patients benefit from this strategy. Therefore, there is an urgent need to develop additional neuroprotective strategies that may, whether or not combined with hypothermia, provide an even better neurological outcome [1].

A promising approach for studying neuroprotection is investigating the physiological phenomenon of preconditioning, which was first described in the brain in 1964 [2]. The underlying mechanisms governing this phenomenon have not been fully elucidated yet. Insight into these mechanisms could provide us with directions for future neuroprotective strategies. Genomic reprogramming could explain many of these mechanisms [3] and with genome-wide micro-array technology it is now possible to investigate this neuroprotective reprogramming in experimental models.

So far, seven studies have investigated large scale gene expression with micro-array techniques after preconditioning in the newborn or adult brain [4-10]. Several of these studies did not adopt a genome-wide approach but used limited and therefore biased transcripts on the array. Furthermore, different experimental paradigms were used and, considering that no paradigm incorporated the fetal-to-neonatal transition, none of these truly resembled the global impact of perinatal asphyxia. Therefore, unique physiological mechanisms specific to the time of birth are missed, although these may play an important role in the development of post-asphyctic brain injury and/or neuroprotection.

Here we present a whole genome micro-array study in a previously validated model where we combined a global perinatal asphyctic insult at the time of birth, with fetal asphyctic (FA) preconditioning on embryonic day 17 (E17). We previously reported that animals subjected to fetal preconditioning on E17, 4 days before suffering severe perinatal asphyxia at birth, had better survival and less brain apoptosis postnatally [11]. Moreover, preconditioned animals performed similar to control animals in behavioral testing at 6 months of age [12]. Consequently, the time-point we chose to investigate the genomic response in the brain is E21, which is just before birth and 96 hours after FA. In these fetal brains we expect to find the neuroprotective mechanisms that are in place perinatally. Accordingly, we hypothesize that the preconditioned animals show a neuroprotective gene expression pattern different from control animals. Furthermore, we chose to take our micro-array data-analysis beyond the single-gene approach, and subjected our data to Gene Set Enrichment Analysis in order to derive results with maximum biological relevance [13].

## Results

### Changes in mRNA expression following preconditioning

Whole genome micro-array technology was used to evaluate differential gene expression in preconditioned animals compared to controls, 96 hours after FA preconditioning. Single-gene analysis with the Bioconductor Limma package yielded 88 transcripts that were differentially expressed with a p-value <0.01, fold changes ranged from 0.69 to 1.43 (see Additional file1). We found 53 transcripts that were down-regulated and 35 transcripts that were up-regulated after FA preconditioning. The majority of down-regulated genes are involved in signal transduction (*expi, gjb6, itgbl1, itpka, lims2, slc22a2, slc35f4*), synaptic transmission (*hpcal4, htr1b, myrip, tomt, vamp5*), and metabolism (*abhd3, adh1, aplnr, osbp2, pdia5*). Most up-regulated genes exert their function in the cell nucleus (*dbx2, elf2, emx2, id1, prim1, rbmx, ste2, zfp862*), regulating transcription factors and proteins involved in DNA and RNA binding. Furthermore, we observed down-regulation of *dbx2, nav1, and mpzl2* as well as up-regulation of *fgfr4, leptin* and *smad6* which seem to be involved in brain development.

**Figure 1:**
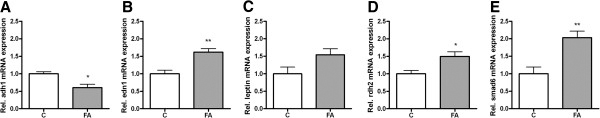
**RT-qPCR validation of micro-array results in whole hemisphere. A**-**E** show RT-qPCR validation results in whole cerebral hemisphere represented as mean + SEM normalized to control (n = 4-6). *Adh1* (p < 0.05), *Edn1* (p < 0.01), *Rdh2* (p < 0.05) and *Smad6* (p < 0.01) demonstrate a significant difference. *Leptin* shows a trend towards significance with a p = 0.07. (White bars = Control, grey bars = Fetal Asphyctic Preconditioning, significance was tested with unpaired two-tailed Student’s t-test: * = p < 0.05, ** = p < 0.01).

**Figure 2:**
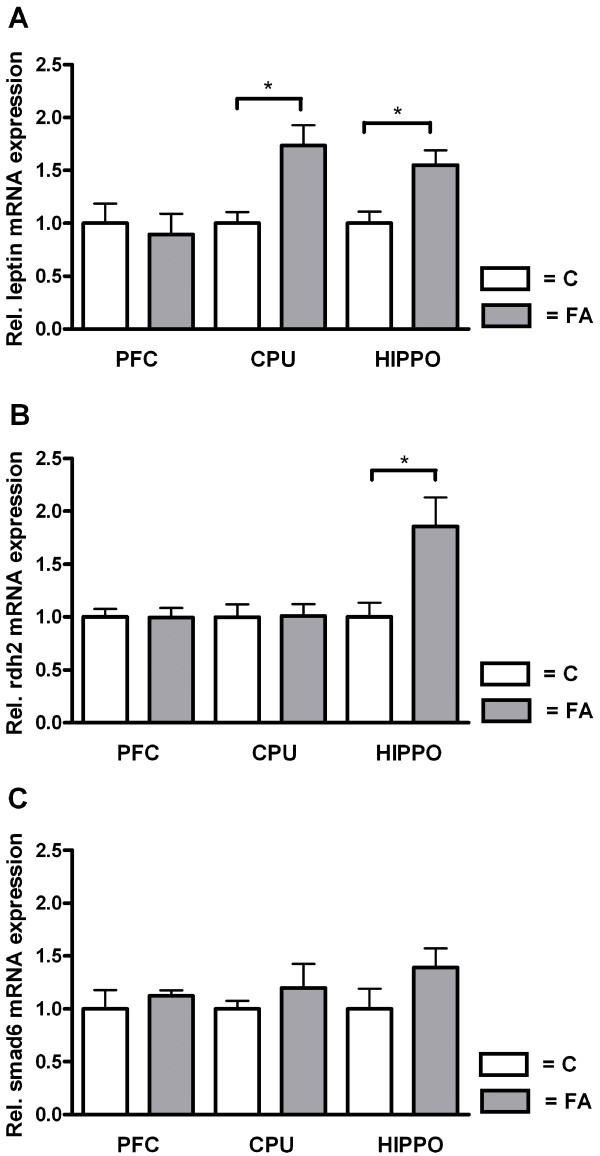
**RT-qPCR validation of micro-array results in brain regions. A**-**C** show RT-qPCR validation results in prefrontal cortex (PFC), caudate-putamen (CPU), and hippocampus (HIPPO) represented as mean + SEM normalized to control (n = 4-6). *Leptin* reveals a significant difference in CPU and HIPPO (p < 0.05), *Rdh2* demonstrates only a significant difference in HIPPO (p < 0.05), and *Smad6* does not show significant differences in the investigated brain regions. (White bars = Control, grey bars = Fetal Asphyctic Preconditioning, significance was tested with unpaired two-tailed Student’s t-test: * = p < 0.05).

In order to verify micro-array results we randomly chose several genes from the Limma analysis to validate with Real-Time qPCR (RT-qPCR) (see Figure 1A-E). We found a significant difference, confirming our micro-array results, for *adh1* (p = 0.01), *edn1* (p = 0.004), *rdh2* (p = 0.03), and *smad6* (p = 0.005). A trend towards significance was found for *leptin* (p = 0.07). Moreover, RT-qPCR was used to evaluate possible regional differences in expression for *leptin*, *rdh2* and *smad6* (see Figure 2A-C). Analysis of *leptin* mRNA expression revealed a significant up-regulation in the FA group in CPU (p = 0.01) and hippocampus (p = 0.02), and no significant difference in PFC. For *rdh2* we observed no significant differences in PFC and CPU, but we found a significant up-regulation in FA animals in the hippocampus (p = 0.04) Finally, analysis of *smad6* mRNA expression in prefrontal cortex (PFC), caudate-putamen (CPU), and hippocampus revealed no significant difference between control and FA animals.

### Changes in biological pathways: Gene Set Enrichment Analysis

**Figure 3:**
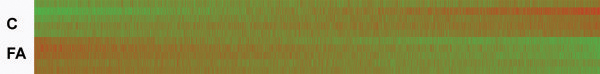
**Heatmap of ranked data-sets for Gene Set Enrichment Analysis.** This heatmap, based on the moderated t-statistics generated with the Bioconductur Limma package, visualizes the differential expression between both phenotypes. Horizontal lines represent the 10 individual arrays, 5 control (C) on top, and 5 fetal asphyctic (FA) preconditioning in the bottom, with red indicating high expression and green indicating low expression. Vertical lines represent the expression of the individual genes. Expression in phenotype FA is higher than C on the top of the ranked list (left), expression in phenotype C is higher than FA at the bottom of the ranked list (right).

In order to derive results with maximum biological relevance we decided to subject our data to a pathway based approach. The method we used was Gene Set Enrichment Analysis (GSEA) for which the entire data set was ranked according to moderate t-statistics (see Figure 3).

GSEA analysis revealed that, out of a total of 737 gene sets, 10 gene sets were significantly enriched (FDR q-value <0.05) in the FA group and therefore up-regulated in the preconditioned animals (see Additional file 2). Also, 19 gene sets were significantly enriched (FDR q-value < 0.05) in the control animals and therefore down-regulated in the preconditioned animals (see Additional file 3). The majority of down-regulated gene sets play a role in signal transduction, and the remaining gene sets are important in synaptic transmission. The majority of up-regulated gene sets have their gene products located in the cell nucleus, and the remaining gene sets are important for ribosomal structure.

To identify which genes contributed most to the enrichment score within different gene sets, we performed a Leading Edge Analysis in the GSEA environment. In such an analysis enriched gene sets are examined for genes that occur before the maximum of the running enrichment signal, because these genes are the core of the gene set that drive the enrichment signal [13]. Our Leading Edge Analysis revealed that there were 367 individual transcripts present in the leading edge of up-regulated gene sets, and 377 transcripts in the down-regulated gene sets (all annotated genes can be found in Additional files 2 and 3). The genes in up-regulated gene sets include many histone clusters, ribosomal proteins and transcription factors, but interestingly also several key epigenetic players such as: *hdac 1, hdac2, hdac3, myst3, mecp2, pcgf2, dnmt1 and dnmt3l*. Among the Leading Edge genes in down-regulated gene sets were many different neurotransmitter receptors such as: glutamate receptors, GABA receptors, serotonin receptor, and a dopamine receptor. However, initial GSEA analysis showed that ‘regulation of glutamate secretion’ was the only significant gene set specific to one neurotransmitter (NES 1.94, FDR q-value 0.019). Besides genes related to neurotransmitter receptors we also found many genes that play a role in the pre-synaptic phase of neurotransmission. These genes (*cplx2; cplx1; pclo; slc18a2; snap25; snap91; stxbp2; stx3; syn1-2; syt1-2; syt10; sv2b; vamp2*) play different roles in the process of synaptic vesicle exocytosis such as: vesicular transport, docking, priming, and ultimately vesicular fusion.

## Discussion

Here we present the first whole genome expression data in fetal brain tissue after fetal preconditioning. In concordance with our hypothesis we found that preconditioned animals have a gene expression pattern that is different from control animals. We chose to take our micro-array data-analysis beyond the single-gene approach, and subjected our data to Gene Set Enrichment Analysis in order to derive results with maximum biological relevance. Our most interesting finding is that up-regulated gene expression seems to involve epigenetic mechanisms.

### Gene Set enrichment analysis

Analyzing micro-array results is typically done by comparing genes on a gene-by-gene basis and assessing if they are differentially expressed between experimental groups. In this approach the focus is on the genes that show the largest difference in expression between the experimental groups. However, this approach has some vital limitations. Most importantly it assumes that all genes act independently of one another, although biologically this is not the case. It is well known that biological processes often affect sets of genes that act simultaneously. Therefore, a small increase in all genes that belong to a certain pathway is likely to be more biologically relevant then a high increase in a single gene in that pathway [13]. Consequently, we chose Gene Set Enrichment Analysis which is a pathway-oriented approach, in order to obtain results that resemble physiological circumstances. Interestingly, GSEA results revealed a similar, but more extensive perspective than our Limma analysis results. Both indicate that the most up-regulated genes were involved in processes within the cell nucleus, and that the most down-regulated genes were involved in signal transduction and synaptic transmission. However, GSEA provided more information on the pathways that are involved.

### Involvement of epigenetic mechanisms

With Limma analysis we found that the majority of up-regulated transcripts have gene products located in the cell nucleus. Their function is related to DNA binding, replication, and transcription.

Further GSEA analysis on our data revealed the majority of significantly enriched gene sets in the preconditioned animals are also mainly concerned with the cell nucleus, such as chromatin, the nucleosome, and histones. Ultimately, investigation of the Leading Edge Genes in these gene sets revealed several well known epigenetic players involved in histone acetylation (*hdac1, hdac2, hdac3, myst*) and DNA methylation (*mecp2, dnmt1, dnmt3l*). It is well known that *mecp2* interacts with methylated DNA and, together with histone deacetylases, is able to cause transcriptional repression [14]. Since we observe marked down-regulation in several other functional categories we now wonder if the observed down-regulation is a consequence of the up-regulation of epigenetic players. Although the involvement of genes that have their gene products in the cell nucleus, such as DNA binding proteins or proteins involved in cell cycle control, was previously demonstrated in preconditioning studies, the pathways involved were not clear and in particular the link to key epigenetic players has not been previously described in a whole transcriptome approach [5,6,8-10]. In a recent review epigenetic changes were suggested to be the ‘master switch’ for activating neuroprotective pathways after preconditioning [15]. Even though there is growing evidence for a role of epigenetic mechanisms in neuroprotection, the evidence today is contradictory regarding the mechanisms of action. For example, different types of HDAC family members seem to exert different functions in mediating neuroprotection. While inhibitors of HDACs can reduce brain injury in an adult ischemia model, activation of NAD + dependent HDACs was shown to be protective in a preconditioning model [16,17]. In addition, *mecp2* expression has previously been linked to preconditioning induced neuroprotection in a mouse model of focal ischemia, whereas a *dnmt1* knockout model showed significantly smaller infarct size after stroke [18,19]. Our results presented here lend support for more in-depth research of epigenetic mechanism involved in neuroprotection.

### Synaptic transmission

A well known cause of brain damage after perinatal asphyxia is the excessive release of the major excitatory amino acid glutamate, leading to massive Ca^2+^ influx and ultimately neuronal death [20]. In the present study we observed down-regulation of several genes related to exocytosis in the preconditioned animals, such as ‘Myosin VIIA and Rab interacting protein’ (*myrip*), which is an integral element of vesicle docking machinery. Recently it was demonstrated to function as a scaffolding protein that links protein kinase A to the exocytosis machinery [21]. The results of our GSEA pathway-based analysis point even stronger in the direction of neurotransmission with 5 significantly down-regulated gene sets related to neurotransmission or synaptic vesicles in the preconditioned animals. Although Leading Edge Analysis suggests several different neurotransmitter pathways to be down regulated after preconditioning, we found that the only significantly enriched gene set specific to one neurotransmitter, was the Gene Ontology term: ‘regulation of glutamate secretion’. This indicates that, among the different down-regulated processes of synaptic transmission, the down-regulation of glutamate signaling is most prominent in preconditioned animals. Logically, this could make preconditioned animals less vulnerable for excitotoxic damage in a subsequent perinatal asphyctic insult, and therefore contribute to the previously observed ischemic tolerance in these animals. Also, glutamate receptor antagonists have been tested for their neuroprotective properties in hypoxia-ischemia [22]. Furthermore, a long-lasting reduced expression of the glutamate receptor NR1 subunit was previously described in neonatal rats after fetal hypoxia-ischemia [23]. On the other hand we know that physiologic changes in glutamate receptor levels are an important part of brain maturation because of glutamate-mediated neuroplasticity [24]. Therefore, it is possible that this preconditioning induced change in glutamate signaling interferes with normal brain development. Further studies are needed to establish if there is a negative effect of down-regulated neurotransmission on neonatal brain development.

### Signal transduction

Both Limma and GSEA reveal a marked down-regulation in this functional category, with the exception of posphodiesterase 9a (*pde9a*) and *slc22a13* up-regulation in Limma analysis. From all phosphodiesterases *pde9a* has the highest affinity for cyclic guanosine monophosphate (cGMP).In the brain cGMP synthesis is increased after NMDA-receptor activation, on the other hand *pde9a* is known to modulate the response to dopaminergic, serotonergic and cholinergic neurotransmission [25,26]. The asphyctic preconditioning stimulus is likely to have caused NMDA-receptor activation due to excessive glutamate release, and possibly activated other neurotransmitter receptors as well, which could explain the observed up-regulation in *pde9a*.

GSEA revealed a marked down-regulation of many gene-sets related to signal transduction: PFAM Ion channel family, PFAM organic anion transporter polypeptide, GO ion channel activity, GO voltage gated ion channel activity, GO cation channel activity, GO metal ion transmembrane transporter activity, KEGG calcium sigalling pathway, KEGG long term potentiation, KEGG salivary secretion, KEGG gastric acid secretion, KEGG pancreatic secretion, and finally the Biocarta Nos1 pathway. In literature, the down-regulation of genes related to signal transduction after preconditioning has been compared to neuroprotective strategies in hibernation [6]. Moreover, ion channels have been implicated in excitotoxicity associated mitochondrial Ca^2+^ overload, cell energy failure and ultimately cell death [27]. A decrease in ion channels after preconditioning could be protective in a subsequent asphyctic insult by preventing Ca^2+^ overload.

### Brain development

We found an up-regulation of *leptin*, SMAD family member 6 (*smad6*), and fibroblast growth receptor 4 (*fgfr4*). *Leptin* is in involved in regulation of neural function, development and survival. Moreover, in a recent stroke study it was shown to exert neuroprotective effects when administered systemically [28]. Smad6 is one of the signaling proteins in the TGF-ß superfamily, although it has a different structure than the other SMAD proteins and is thought to be a negative regulator of TGF-ß signaling [29]. TGF-ß is a cytokine with a wide range of functions, possibly including neuroprotection [30]. However, recently it was shown that inhibition of TGF-ß-activated kinase (*TAK1*) is neuroprotective in stroke by preventing apoptosis via the Jun kinase (*JNK*) pathway [31]. By linking increase *smad6* transcription to negative regulation of TGF-ß, and neuroprotection induced by inhibition of *TAK1*, the observed up-regulation of s*mad6* could indicate a preconditioning-activated neuroprotective mechanism. *Fgfr4* was up-regulated in preconditioned animals. It binds several members of the Fgf-family, of which *fgf2* is probably the most potent neurotrophic factor [32]. We observed an up-regulation of e*mx2* which is a homeobox gene required for hippocampal development. In a primate study *emx2* was suggested to be a putative controller of progenitor cell fate in the hippocampus[33]. Therefore, up-regulation of *emx2* could help restore neuronal production in the hippocampus after an ischemic event. For another homeobox gene (*dbx2*) we observed down-regulation in preconditioned animals. *Dbx2* is known to play an important role in neural patterning and differentiation but was never before implicated in ischemia or neuroprotection [34].

### Metabolism

Limma analysis and subsequent qPCR validation demonstrated up-regulation of retinol dehydrogenase 2 (*rdh2*), especially in CPU and hippocampus. Rdh2 is a short chain dehydrogenase/reductase isoenzyme that catalyzes the first step of retinoic acid synthesis [35]. Retinoic acid is known to modulate gene expression and to exert cytoprotective effects. Recently it was suggested to reduce ischemia-induced cerebral damage by an anti-inflammatory mechanism [36].

### Ribosomal proteins

With GSEA we observed an up-regulation of PFAM and KEGG pathways concerning ribosomal proteins. Asphyxia has long been known to disrupt ribosomal structure and function which leads to diminished protein synthesis and thereby contributes to early phase cell death. Moreover, preconditioning has been shown to facilitate the recovery of protein synthesis following lethal ischemia in preconditioned gerbils [37]. Recently, pathway analysis in a micro-array study of ischemic preconditioning demonstrated an up-regulation of ribosomal pathways following a severe ischemic insult [4]. This seems similar to our findings, however we demonstrate that the up-regulation in ribosomal pathways is already present after the preconditioning stimulus alone, so before the deleterious insult has even occurred, possibly preparing the brain for disruption of ribosomal structure during a subsequent asphyctic insult.

### Whole cerebral hemisphere expression and regional differences

For these micro-array experiments we used complete cerebral hemispheres, thereby including different brain regions that might have counteracting gene expression, as was previously described in the adult mouse brain after hypoxic preconditioning [9]. The observed differences in gene expression across different brain regions might be related to their phylogenetic age or metabolic activity. However, in order to determine the genomic reprogramming mechanisms that have an overall effect in the brain we chose to subject the complete cerebral hemisphere to micro-array investigation. Following confirmation of micro-array results for several genes with RT-qPCR in complete hemispheres, we determined the expression of these genes in different brain regions and indeed we observed some differential regional expression. Nevertheless, our aim was to investigate overall mechanisms of asphyctic preconditioning in the brain.

## Conclusions

This study is the first to investigate whole genome transcription in the fetal brain after asphyctic preconditioning and we present several interesting findings. Firstly, we confirmed altered gene expression after FA preconditioning. The majority of differentially expressed genes are down-regulated, which makes our findings consistent with previous research where different model approaches were used, and at the same time lends validity to previous findings [6,7]. In addition we describe several genes that were not previously linked to preconditioning and/or neuroprotection. Secondly, by adding GSEA to our initial single gene analysis we were able to derive results with maximum biological relevance. Finally, we found that the majority of up-regulated genes have gene products located in the cell nucleus, and GSEA pathways clearly indicate that epigenetic mechanisms play a role in preconditioning induced neuroprotection. This is the first micro-array study to demonstrate such a clear link between neuroprotection and epigenetic mechanisms, however, additional research into these mechanisms is required.

## Methods

### Animals

All experiment protocols were approved by the Animal Ethics Board of Maastricht University according to Dutch governmental regulations.

Adult Sprague–Dawley rats (body weight 250-320 g), obtained from Charles River (France), were kept under standard laboratory conditions with food and water given ad libitum, 21 ± 2°C environment temperature, a 12 h light/dark schedule (lights on at 07:00 h) and background noise provided by radio. Breeding was carried out in our own animal facility to prevent stress resulting from transportation during pregnancy. To facilitate mating a female was housed together with a male for 1 night in a breeding cage. The duration of gestation was determined by the observation of a mucus plug. Exclusively male offspring were used in this study, because both morphological and behavioral evidence show a differential vulnerability to a birth insult in males versus females. A greater impact is seen in the male gender, possibly due to the difference in circulating sex hormones compared to females [38].

### Animal model

Dams were anesthetized by isoflurane (4% induction and 1.5% maintenance) on E17 and subjected to a midline laparotomy. Next, FA preconditioning was induced by completely clamping both uterine and both ovarian arteries with removable clamps for 30 minutes. Thereafter, dams were sutured up to recover in their home-cage. These procedures were performed inside a closed incubator to maintain normothermia and 75% air humidity. Wild-type dams were used as control mothers.

### Tissue preparation

**Figure 4:**
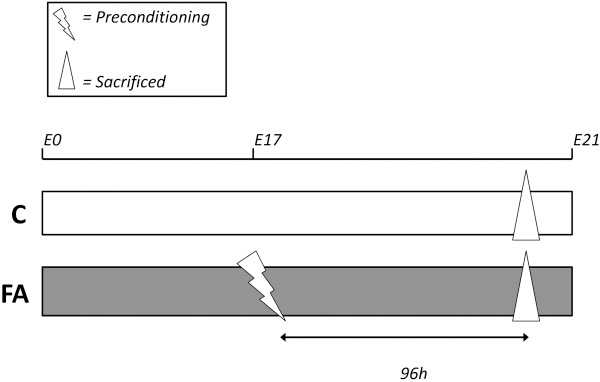
**Experimental design.** Two experimental groups were used: Control (C) and Fetal Asphyctic (FA) preconditioning. On embryonic day 17 (E17) FA animals were subjected to a 30-minute preconditioning stimulus by clamping the uterine circulation, and 96 hours later both experimental groups were sacrificed. Control animals are wild-type.

FA and control pups were delivered on E21 by Caesarean section and immediately decapitated. Control animals have not undergone any intervention prior to birth (see Figure 4). To prevent litter effects only one pup per dam was used for micro-array analysis, with a total of five males per condition. After removing the cerebellum left hemispheres were dissected and submerged in RNA stabilizing reagent (Qiagen, Benelux BV, Venlo, The Netherlands). Samples were kept at 4°C for four days, before being snap frozen in liquid nitrogen, and ultimately stored at −80°C. For RT-qPCR analysis a maximum of two pups per litter were used, with a total of five pups per condition. Right hemispheres were dissected, then snap frozen in liquid nitrogen, and ultimately stored at −80°C. Additionally, in six males per condition we dissected three different brain regions: prefrontal cortex (PFC), caudate-putamen (CPU), and hippocampus for analysis of regional expression by RT-qPCR. Dissection of these brain regions was performed in situ under 4x magnification immediately after sacrificing the pups.

### RNA-isolation

For micro-array analysis total RNA extraction and purification were performed on mini RNeasy columns (Qiagen Benelux BV, Venlo, The Netherlands), according to the manufacturer’s instructions. Quantity and purity of total RNA was determined by spectrophotometer analysis using the Nanodrop ND-1000 (Thermo Fisher Scientific Inc., Waltham, USA). Only samples with a 260/280 ratio between 1.8 and 2.1, and a 260/230 ratio between 1.5 and 2.0 were selected for micro-array analysis. Additionally, RNA quality measurements were performed with Bioanalyzer 2100 (Agilent Technologies Netherlands B.V.). Samples with an RNA integrity number (RIN) below 8 were excluded.

For RT-qPCR total RNA was extracted with Trizol® reagent (Invitrogen, Paisley Scotland, UK) according to manufacturer’s instructions. Next, cDNA was generated with RevertAid First Strand cDNA synthesis kit (Fermentas GMBH, St. Leon-Rot, Germany).

### RT-qPCR

RT-qPCR reactions were carried out using SYBR green PCR master mix and the LightCycler 480 (Roche Diagnostics, Almere, The Netherlands). To evaluate relative expression we used *Gapdh, Hprt, and ß-actin* as internal controls. Sequences of primers used can be found in Table 1.

**Table 1:** Primer design for RT-qPCR

	**Forward**	**Reverse**
**GAPDH**	5’-CTCCCATTCTTCCACCTTTG	5’-ATGTAGGCCATGAGGTCCAC
**HPRT**	5’-TTGCTGGTGAAAAGGACCTC	5’-TCCACTTTCGCTGATGACAC
**ß-actin**	5’-TTGCTGACAGGATGCAGAAG	5’-TGATCCACATCTGCTGGAAG
**Adh1**	5’-CCTTCCCGGTTTCTGACTCC	5’-TCTCACGGAAAGCTTGCACA
**Edn1**	5’-CACCGTCCTCTTCGTTTTGC	5’-TGGAAAGCCACAAACAGCAG
**Leptin**	5’-GTTCCTGTGGCTTTGGTCCT	5’-CTGGTGACAATGGTCTTGATG
**Rdh2**	5’-CTGGATGTGAACCTGTTGGG	5’-ACCACAAAAAGACAGTCGGC
**Smad6**	5’-ACCCCTACCACTTCAGCC	5’-GGTCAGGAGGAGACAGCC

### Micro-array analysis

Using the Ambion WT Expression Kit, per sample, an amount of 100 ng of total RNA spiked with bacterial poly-A RNA positive controls (Affymetrix Inc., Santa Clara, USA) was converted to double stranded cDNA in a reverse transcription reaction. Next the sample was converted and amplified to antisense cRNA in an *in vitro* transcription reaction which was subsequently converted to single stranded sense cDNA. Finally, samples were fragmented and labeled with biotin in a terminal labeling reaction according to the Affymetrix WT Terminal Labeling Kit. A mixture of fragmented biotinylated cDNA and hybridization controls (Affymetrix Inc., Santa Clara, USA) was hybridized on Affymetrix GeneChip Rat Gene 1.0 ST Arrays followed by staining and washing in a GeneChip® fluidics station 450 (Affymetrix Inc., Santa Clara, USA) according to the manufacturer’s procedures. To assess the raw probe signal intensities, chips were scanned using a GeneChip® scanner 3000 (Affymetrix Inc., Santa Clara, USA). According to MIAME requirements data were submitted the NCBI GEO database, and are available under accession number: GSE42676.

### Gene Set enrichment analysis (GSEA)

For GSEA, a total of 737 rattus norvegicus gene sets were assembled, including 196 KEGG pathways (release 59.0), 81 Biocarta pathways (accessed August 18^th^ 2011), 184 Gene Ontology terms (AmiGO version 1.8), and 276 Pfam protein families database (Pfam 25.0). Each gene-set contained a minimum of 15 genes and a maximum of 500 genes in accordance with GSEA recommendations. The analysis was conducted using the GSEA software v2.07, provided by the Broad Institute (Cambridge, MA, USA) [13].

### Statistics

For RT-qPCR all data were distributed normally as tested with Kolmogorov-Smirnov test. Statistical significance was tested with the unpaired, two-tailed Student’s t-test. Results are presented as means + SEM, normalized to control and p-values <0.05 were considered statistically significant.

Analysis of the micro-array data was performed in the R programming environment (version 2.12.0), with the packages developed within the Bioconductor project [39]. The analysis was based on the RMA expression levels of the probe sets. Differential expression was assessed with the Limma package using moderated t-statistics [40]. Results are presented as fold changes and p-values < 0.01 were considered statistically significant.

For GSEA the micro-array dataset was pre-ranked using moderated t-statistics [40]. A gene set enrichment score (ES) was calculated based on the Kolmogorov-Smirnov statistic and for each gene set the ES was normalized to account for difference in gene set size. Finally, a false discovery rate (FDR) was calculated relative to the normalized enrichment score (NES) values to determine the probability of type I errors. To control for multiple testing we used the false discovery rate (FDR) as described by Benjamin and Hochberg [41]. Enriched gene-sets with an FDR q-value <0.05 were selected. Ultimately we performed a ‘Leading Edge Analysis’ in GSEA on significantly enriched gene-sets, to identify the genes that contribute most to the enrichment signal.

## Abbreviations

C: Control; FA: Fetal Asphyctic preconditioning; GSEA: Gene Set Enrichment Analysis; E17: Embryonic day 17; E21: Embryonic day 21; CPU: Caudate-putamen brain region; PFC: Prefrontal cortex brain region; FDR: False Discovery Rate; KEGG: Kyoto Encyclopedia of Genes and Genomes; GO: Gene Ontology.

## Competing interests

The authors declare that they have no competing interests (both financial and non-financial).

## Author’s contributions

KC participated in design of the study, carried out the animal experiments and RNA extraction, performed Gene Set Enrichment Analysis, and drafted the manuscript. JV and AG participated in study design and coordination and helped to draft the manuscript. JS carried out the qPCR analysis. LZ and ES participated in study design and helped to draft the manuscript. All authors have read and approved the final manuscript.

## Supplementary Material

Additional file 1 **Differentially expressed genes in preconditioned animals 96 h after fetal asphyctic preconditioning (FA).** P-value <0.01 was considered statistically significant. No cut-off was used for fold change. Click here for file

Additional file 2 **Gene Set Enrichment Analysis (GSEA) results; up-regulated gene sets in preconditioned versus control animals 96 h after fetal asphyctic preconditioning (n = 5).** FDR q-value <0.05 was considered statistically significant. Click here for file

Additional file 3 **Gene Set Enrichment Analysis (GSEA) results; down-regulated gene sets in preconditioned animals versus control animals 96 h after fetal asphyctic preconditioning (n = 5).** FDR q-value <0.05 was considered statistically significant. Click here for file
